# Comparative transcriptome analysis of Indian domestic duck reveals candidate genes associated with egg production

**DOI:** 10.1038/s41598-022-15099-5

**Published:** 2022-06-29

**Authors:** Karippadakam Bhavana, Dustin J. Foote, Krishnamoorthy Srikanth, Christopher N. Balakrishnan, Vandana R. Prabhu, Shanmugam Sankaralingam, Hijam Surachandra Singha, Achamveetil Gopalakrishnan, Muniyandi Nagarajan

**Affiliations:** 1 Department of Genomic Science, School of Biological Sciences, Central University of Kerala, Kasaragod, Kerala 671316 India; 2 Department of Biology, East Carolina University, Greenville, NC 27858 USA; 3 Department of Animal Science, College of Agriculture and Life Sciences, Cornell University, Ithaca, NY 14853 USA; 4 Department of Poultry Science, College of Veterinary and Animal Sciences, Mannuthy, Thrissur, Kerala 680 651 India; 5 ICAR-Central Marine Fisheries Research Institute, Ernakulam North PO, Kochi, Kerala 682 018 India

**Keywords:** Transcriptomics, RNA

## Abstract

Egg production is an important economic trait and a key indicator of reproductive performance in ducks. Egg production is regulated by several factors including genes. However the genes involved in egg production in duck remain unclear. In this study, we compared the ovarian transcriptome of high egg laying (HEL) and low egg laying (LEL) ducks using RNA-Seq to identify the genes involved in egg production. The HEL ducks laid on average 433 eggs while the LEL ducks laid 221 eggs over 93 weeks. A total of 489 genes were found to be significantly differentially expressed out of which 310 and 179 genes were up and downregulated, respectively, in the HEL group. Thirty-eight differentially expressed genes (DEGs), including *LHX9, GRIA1, DBH, SYCP2L, HSD17B2, PAR6, CAPRIN2, STC2,* and *RAB27B* were found to be potentially related to egg production and folliculogenesis. Gene ontology (GO) and Kyoto Encyclopedia of Genes and Genomes (KEGG) pathway analysis suggested that DEGs were enriched for functions related to glutamate receptor activity, serine-type endopeptidase activity, immune function, progesterone mediated oocyte maturation and MAPK signaling. Protein–protein interaction network analysis (PPI) showed strong interaction between 32 DEGs in two distinct clusters. Together, these findings suggest a mix of genetic and immunological factors affect egg production, and highlights candidate genes and pathways, that provides an understanding of the molecular mechanisms regulating egg production in ducks and in birds more broadly.

## Introduction

**Figure 1:**
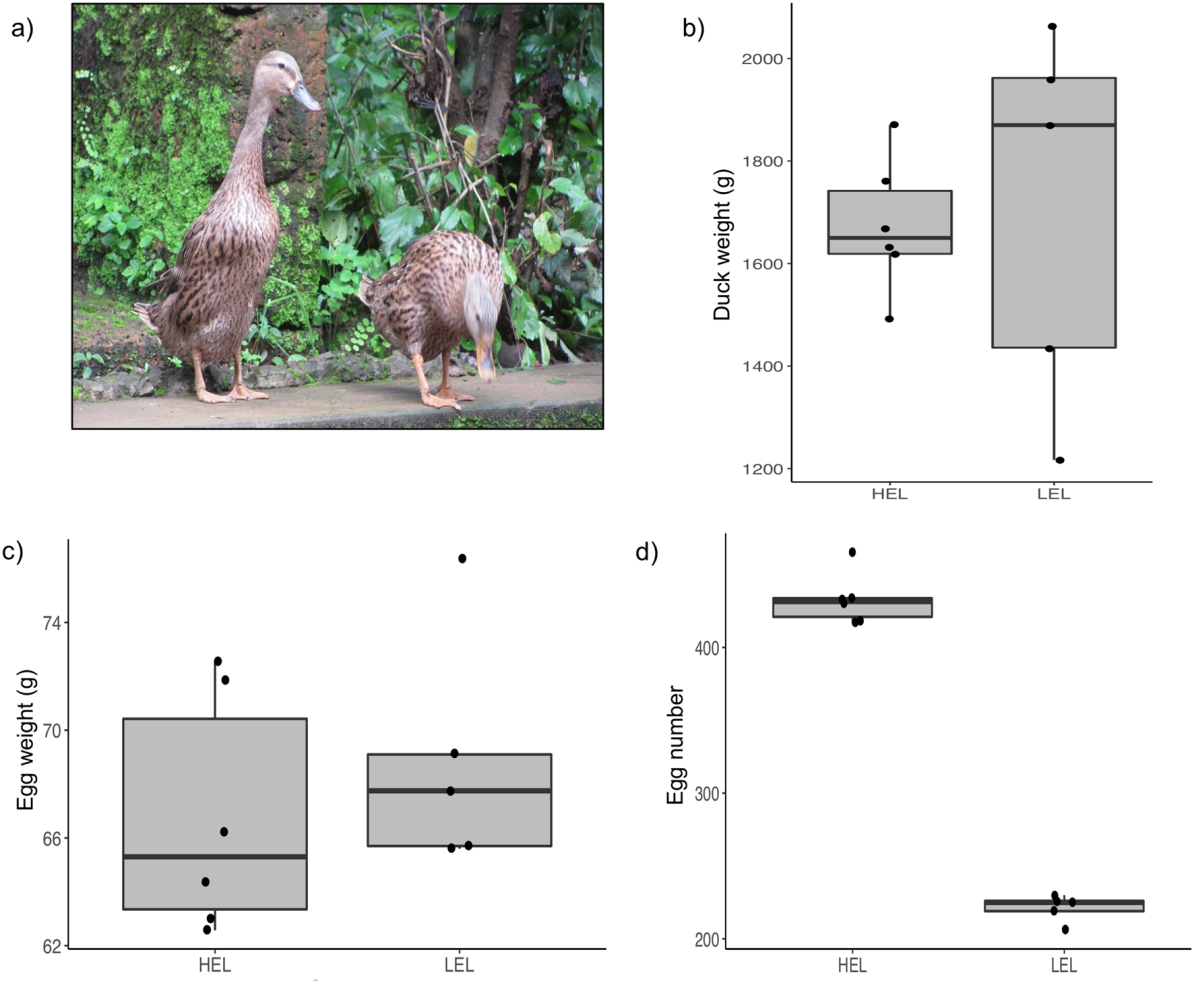
The phenotypic characteristics of HEL and LEL ducks. (**a**) Chara duck (**b**) Box plot showing the body weight distribution of HEL and LEL ducks (**c**) Box plot showing the egg weight distribution of HEL and LEL ducks (**d**) Box plot showing the total number of eggs laid by the HEL and LEL ducks.

The domestic duck (*Anas platyrhynchos*) is an economically important poultry species. Domestic ducks have excellent sustainability because of their high disease tolerance, adaptability, high feed conversion rate and low production cost. There are about 33.51 millions ducks in India, mainly distributed in the north-east and southern parts of the country^1^. A number of duck breeds characterized to varying extents, including, Chara, Chemballi, Pati, Nageswari play a significant role in the livelihood of rural populations^2^. “Chara” is a popular duck breed (Fig. 1a) among farmers mainly due to its high rate of egg production^3^. However, the egg production rate varies quite substantially among individuals and is regulated by several factors^4^. The number of eggs laid by a duck mainly depends on the rate at which the follicles ovulate and mature successfully to a hard-shelled egg, and so the folliculogenesis process determines the egg-laying performance of the duck. Further, studies have shown that, many factors influence the folliculogenesis process, likely the physiology, genes, environment, nutrition, and various metabolic factors of endocrine, paracrine and autocrine functionality, including the gonadotropins, steroid hormones and growth factors.

With the introduction of high throughput sequencing techniques in recent years, understanding complex physiological and biochemical mechanisms have become a possibility. Recently, ovarian transcriptomes of high and low egg producing individuals of Chinese domestic duck, Muscovy duck, goose and chicken have been studied using RNA-Seq^5–11^. These studies have shown that, the reproduction process, comprising folliculogenesis and ovulation, requires many genes to act synergistically. Tao et al.^5^ conducted a comparative study on high and low egg laying Jinding ducks using tissue from the medulla of the ovary, which revealed multiple genes that are involved in, circadian rhythm and estrogen receptor binding. Sun et al.^6^ performed a comparative transcriptome study on Longyan Shan-ma ducks and reported several DEGs and pathways related to ovarian development and egg production. However the potential genes involved in egg production in duck remain unclear and these studies^5,6^ have analysed only a few samples from high and low egg producing ducks. Therefore, it is necessary to investigate more samples and additional breeds to provide a comprehensive view of the molecular mechanism underlying the egg production in ducks.

In the present study we analysed the ovarian transcriptome of high and low egg laying Chara ducks to identify the genes associated with egg production for the improvement of egg production. The results of this study will further the understanding of the gene regulatory mechanisms influencing egg production and laying performance. The genes identified in this study represent candidate genes for marker-assisted selection studies for enhancing egg production in ducks.

## Results

### Phenotypic features

The body weight and egg weight were not significantly different between HEL and LEL ducks (Fig. 1b,c). However, the difference in the total number of eggs laid by HEL and LEL ducks was significant (Fig. 1d). On average HEL ducks laid 212 eggs more than the LEL ducks over 93 weeks.

### Characteristics of duck ovarian transcriptome

**Figure 2:**
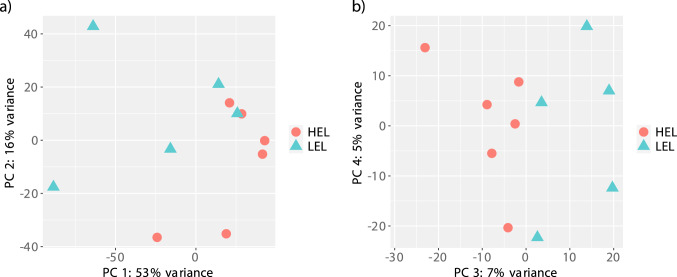
Principal component analysis showing the genetic variation between the HEL and LEL ducks based on transcriptome data. (**a**) PC 1 vs PC 2 and (**b**) PC3 vs PC 4.

To analyse the transcriptome changes of the ovary of HEL and LEL ducks, and to identify the genes associated with egg production, 11 ovarian transcriptomes were sequenced. In total, 806,967,717 million raw reads were generated with an average of 73,360,701 million reads per sample. The number of clean reads ranged from 50,718,335 to 118,504,555 after filtering out ribosomal RNA, adapters, and low quality reads (Table 1). Mapping reports from STAR revealed that 71.4–84.3% of the reads were mapped to the reference duck genome. The mapping rate was comparable to previous transcriptome studies^12,13^. On average 80.5% of reads mapped to annotated genes in the genome. Principal component analysis (PCA) was performed using normalized expression data to understand the grouping and variability between the HEL and LEL duck transcriptomes. Principal component 1 and 2 explained 53% and 16% of the variation in gene expression, respectively (Fig. 2a). Principal component 3 explained another 7% of variation in gene expression (Fig. 2b). We detected expression (> 0 reads in at least one sample) in 14,144 genes (Supplementary Table 1).

**Table 1:** Characteristics of ovarian transcriptome data of high and low egg laying Chara ducks.

Sample ID	Phenotype	Number of raw reads	Number of clean reads	Uniquely mapped reads (%)
HEL-1	High egg layer	92,250,440	91,644,763	78.18
HEL-2		118,736,292	118,504,555	80.55
HEL-3		70,369,639	70,283,917	84.25
HEL-4		73,384,706	73,142,237	82.19
HEL-5		80,183,842	79,977,728	84.33
HEL-6		65,343,505	65,220,462	83.64
LEL-1	Low egg layer	51,045,469	50,718,335	71.35
LEL-2		60,608,642	60,450,319	81.54
LEL-3		56,123,215	55,833,111	73.9
LEL-4		72,853,968	72,764,582	83.9
LEL-5		66,067,999	65,881,587	81.18

### Identification of DEGs

**Figure 3:**
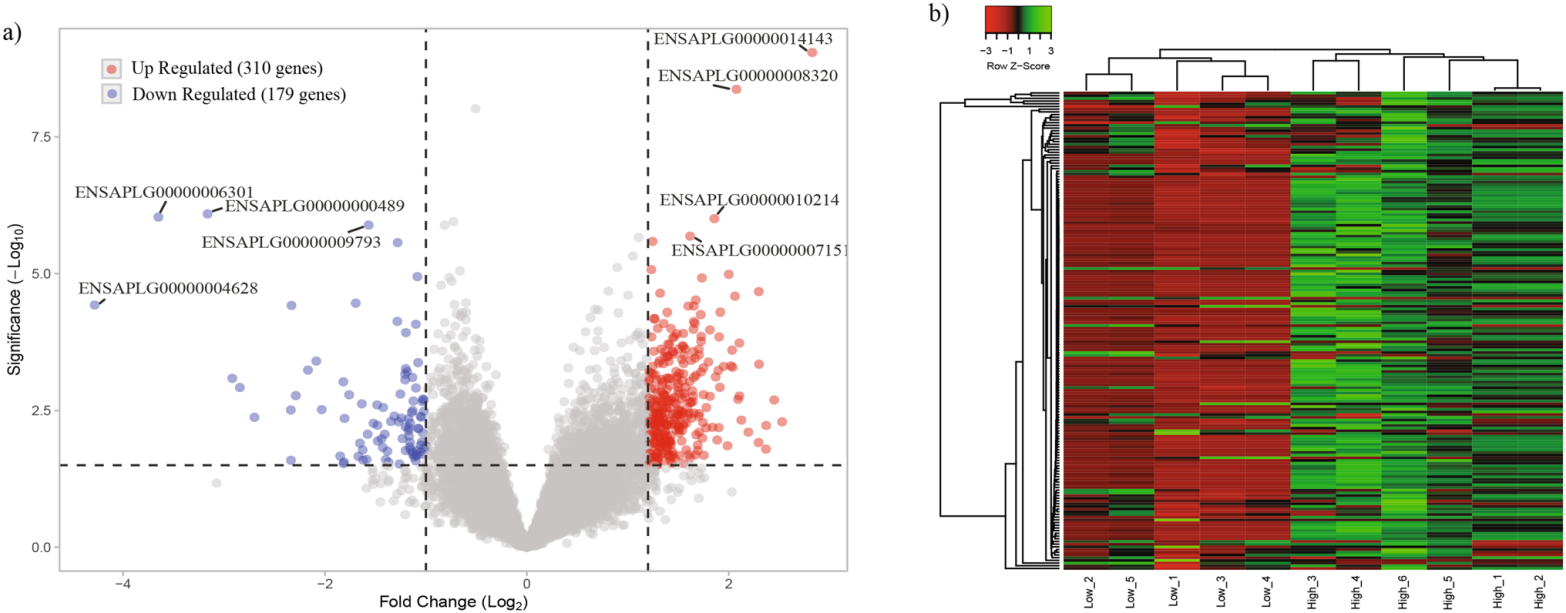
Differential gene expression between HEL and LEL ducks. (**a**) Volcano plot shows the DEGs between the HEL and LEL ducks. Each dot represents a gene. Red, and blue dots indicate upregulated, downregulated DEGs respectively, whereas grey dots indicate non differentially expressed genes. (**b**) Heat map showing the differential gene expression of HEL and LEL ducks. “Red” represents low relative expression level, and “green” represents high relative expression level. Each column and row represent a sample, and a gene respectively.

A total of 489 genes were found to be significantly differentially expressed (FC > 1.5, FDR < 0.1 & *P* < 0.005) between the HEL and LEL ovarian tissues (Supplementary Table 2). Of these 310 and 179 genes were upregulated and downregulated in HEL ducks respectively (Fig. 3a). The top upregulated genes in HEL included *FAM19A4, LHX9, GRIK3, LRFN2, GUCA1B, SLC26A9* and the genes upregulated in LEL (downregulated in HEL) included *P2RX6, TNNT2, ISG15* and *LY96*. Among the top upregulated genes, three were uncharacterized genes (*ENSAPLG00000001875, ENSAPLG00000002685,* and *ENSAPLG00000002064*) and among the top downregulated genes, the top four genes were uncharacterized (*ENSAPLG00000004628, ENSAPLG00000006301, ENSAPLG00000000489,* and *ENSAPLG00000004923*). Hierarchical clustering of the DEG’s showed that, the samples clustered by the group (HEL and LEL) and the DEGs clustered into two major clusters (Fig. 3b). Further, the putative functions of the DEGs were analysed using blast, against the GenBank and UniProt databases, and 38 genes were found to be associated with egg development, folliculogenesis and reproductive functions (Table 2). These included *GRIK3, GRIA1, SYCP2L, KPNA7* and *DBH,* which were enriched in HEL and *P2RX6, CCL5, LY96, CCL3* and *WIF1* were enriched in LEL (downregulated in HEL).

**Table 2:** The list of differentially expressed genes associated with egg production.

S. no.	Gene	Description of the gene	Log2FC	Adjusted P-value
1	*LHX9*	LIM homeobox 9	2.08	3.01E − 05
2	*GRIA1*	Glutamate ionotropic receptor AMPA type subunit 1	1.9	0.04987166
3	*DBH*	Dopamine beta-hydroxylase	1.62	0.00280697
4	*TBPL2*	TATA-Box Binding Protein Like 2	1.57	0.03274777
5	*SYCP2L*	Synaptonemal complex protein 2 like	1.49	0.02517027
6	*KPNA7*	Karyopherin subunit alpha 7	1.39	0.03795863
7	*MOS*	MOS Proto-Oncogene, Serine/Threonine Kinase	1.36	0.03290466
8	*ZAR1L*	Zygote Arrest 1 Like	1.35	0.04379806
9	*TAT*	Tyrosine Aminotransferase	1.32	0.01245165
10	*HSD17B2*	Hydroxysteroid 17-beta dehydrogenase 2	1.31	0.04363603
11	*SLC13A5*	Solute Carrier Family 13 Member 5	1.24	0.04639574
12	*PARD6A/ PAR6*	Par-6 family cell polarity regulator alpha	1.23	0.025425
13	*SGCZ*	Sarcoglycan zeta	1.19	0.03947891
14	*BCAS1*	Brain enriched myelin associated protein 1	1.14	0.04639574
15	*CLDN1*	Claudin 1	1.13	0.04659984
16	*CAPRIN2*	Caprin family member 2	1.02	0.027232
17	*STC2*	Stanniocalcin 2	0.89	0.00731302
18	*PLK3*	Polo Like Kinase 3	0.83	0.0412367
19	*RPS6KA2*	Ribosomal protein S6 kinase A2	0.81	0.0121187
20	*CITED4*	Cbp/p300 interacting transactivator with Glu/Asp rich carboxy-terminal domain 4	0.71	0.01707836
21	*RAB27B*	RAB27B, member RAS oncogene family	0.71	0.02469264
22	*SLC16A1*	Solute carrier family 16 member 1	0.63	0.01865937
23	*ATP1B4*	ATPase Na+/K+ transporting family member beta 4	− 0.60	0.01707836
24	*PODN*	Podocan	− 0.61	0.01919122
25	*TSPO*	Translocator protein	− 0.62	0.04832802
26	*GALNT1*	Polypeptide *N*-acetylgalactosaminyl transferase 1	− 0.64	0.01707836
27	*SERPINE2*	Serpin family E member 2	− 0.68	0.04842376
28	*BSG*	Basigin (Ok Blood Group),	− 0.70	0.04800023
29	*LAMP1*	Lysosomal-associated membrane protein 1	− 0.71	0.02199605
30	*FGF7*	Fibroblast growth factor 7	− 0.73	0.00203647
31	*ENPP3*	Ectonucleotide pyrophosphatase/phosphodiesterase 3	− 0.74	0.00893284
32	*MYLIP*	Myosin regulatory light chain interacting protein	− 0.77	0.00809806
33	*CCL5*	C–C motif chemokine ligand 5	− .78	0.26600280
34	*ACOX2*	Acyl-CoA oxidase 2	− 0.81	0.02030386
35	*TGIF1*	TGFB induced factor homeobox 1	− 0.85	0.01003298
36	*WIF1*	WNT inhibitory factor 1	− 1.19	0.04987166
37	*LY96*	Lymphocyte antigen 96	− 1.28	0.01982797
38	*P2RX6*	Purinergic receptor P2X 6	− 2.17	0.04842376

### qPCR verification of DEG

qRT-PCR was performed on seven randomly selected DEGs such as *LRFN2, RIN2, FAM19A4, PERP, CCL5, OVCH1* and *LY96*. The qPCR results of these genes were in accordance with the RNA-Seq results, suggesting that the RNA-Seq results are reliable.

### GO classification and functional annotation of DEGs

**Figure 4:**
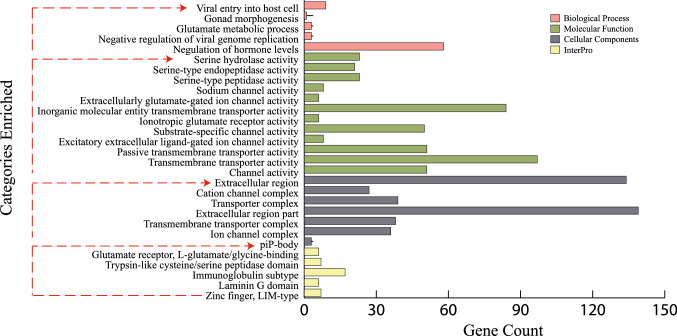
GO enrichment analysis, showing terms significantly enriched with the identified DEG’s between HEL and LEL ducks.

To determine the biological functions of DEGs, we performed over representation analysis with the Gene Ontology and *InterPro* databases. Genes were enriched for 19 Gene Ontology categories (FDR ≤ 0.1; Fig. 4; Supplementary Table 3). Among them, seven categories were enriched for cellular functions, and 12 categories were enriched for molecular functions. Serine hydrolase, serine-type endopeptidase and serine-type peptidase activity were among the highly enriched molecular functions terms. These categories included 24 genes, including Cathepsin family genes (*CTSC*: log2FC = 1.41, FDR = 0.010; *CTSL*: log2FC = 1.34, FDR = 0.017 and *CTSH:* log2FC = 0.83, FDR = 0.032), coagulation factors (*F3*: log2FC = − 0.59, FDR = 0.097; *F10*: log2FC = − 0.625, FDR = 0.07), ovochymase (*OVCH1*: log2FC = 1.34, FDR = 0.099 and *OVCH2*: log2FC = 1.15, FDR = 0.087) and peptidases such as matrix metallopeptidase 13 (*MMP13*: log2FC = − 2.84, FDR = 0.067) and HTRA serine peptidase (*HTRA1*: log2FC = − 0.735, FDR = 0.071). The other prominent category enriched were ion channel functions (e.g., sodium channel activity, 8 genes, *p* = 9.53E − 5, FDR = 5.58E − 2) and the related extracellularly glutamate-gated ion channel activity (6 genes, *p* = 8.99E − 5, FDR = 6.15E − 2). These categories included *GRIA1* (log2FC = 1.895, FDR = 0.049) and *GRIK3* (log2FC = 2.06, FDR = 0.013).

None of the GO categories in the biological process domain was statistically enriched after correcting for multiple hypothesis testing (Supplementary Table 3). These mildly enriched biological process categories, however, highlight genes associated with immunity (e.g., viral entry into host cells (*p* = 9.5E − 5, FDR = 0.338), gonad morphogenesis (*p* = 7.05E − 4 and FDR = 0.476), glutamate metabolic process (*p* = 2.15E − 04, FDR = 3.04E − 01), negative regulation of viral genome replication (*p* = 3.69E − 04, FDR = 4.76E − 01)) and regulation of hormone levels (*p* = 4.67E − 4 and FDR = 4.14E1). Only a single gene, LIM homeobox 9 (*LHX9)* was associated with gonad morphogenesis which was among the most strongly differentially expressed genes (log2FC = 2.07 FDR = 3.01E − 05). *MX1* gene was one of nine genes associated with viral entry into host cells and is also differentially expressed (log2FC = − 1.08, FDR = 0.008). Genes associated with viral entry into host cells and other immune-related patterns tend to be upregulated in LEL ducks (5/9 genes enriched under viral entry into host cell GO term). Moreover, functional classification of DEGs according to protein family domains based on InterPro database revealed that 17 DEGs were enriched under Immunoglobulin subtype. In addition to these, various other genes including, *FAM19A*, dopamine receptor D2 (*DRD2*), Ras and Rab interactor 2 (*RIN2*), *LHX9* that are associated with reproductive developmental process like egg maturation, folliculogenesis and gonad morphogenesis were also differentially expressed (Fig. 4; Supplementary Table 3).

### KEGG pathway enrichment

**Figure 5:**
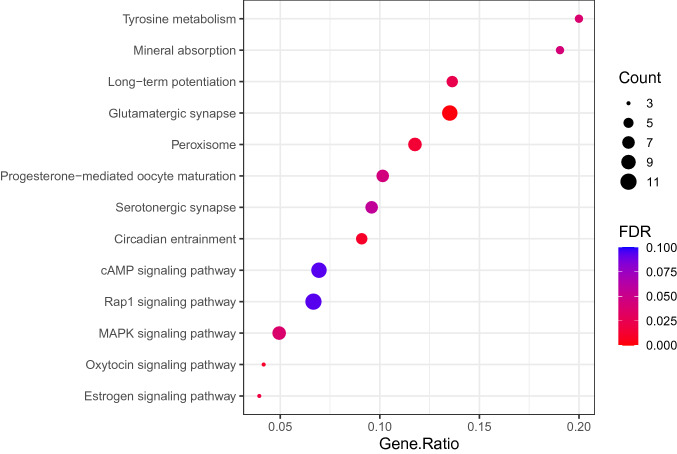
KEGG pathways enriched by the DEGs. The x axis represents the GeneRatio of the enriched genes and y axis shows the names of the enriched pathways. The area of each dot represents the number of enriched genes and the color represents the significance of the FDR value (red represents high significance, while blue represents low).

To obtain additional functional classification and pathway assignments of the DEGs, KEGG pathway enrichment analysis was performed. Several pathways showed enrichment (Fig. 5; Supplementary Table 4) including, Rap1 signaling pathway (hsa04015, adjusted *p* = 0.034, 11 genes), Glutamatergic synapse (hsa04724, adjusted *p* = 0.0016, 10 genes), cAMP signaling pathway (hsa04024, adjusted *p* = 0.093, 10 genes), MAPK signaling pathway (hsa04010, adjusted *p* = 0.037, 9 genes), progesterone mediated oocyte development (hsa04914, adjusted *p* = 0.044, 7 genes), and Circadian entrainment (hsa04713, adjusted *p* = 0.010, 6 genes).

### Protein–protein interaction network

**Figure 6:**
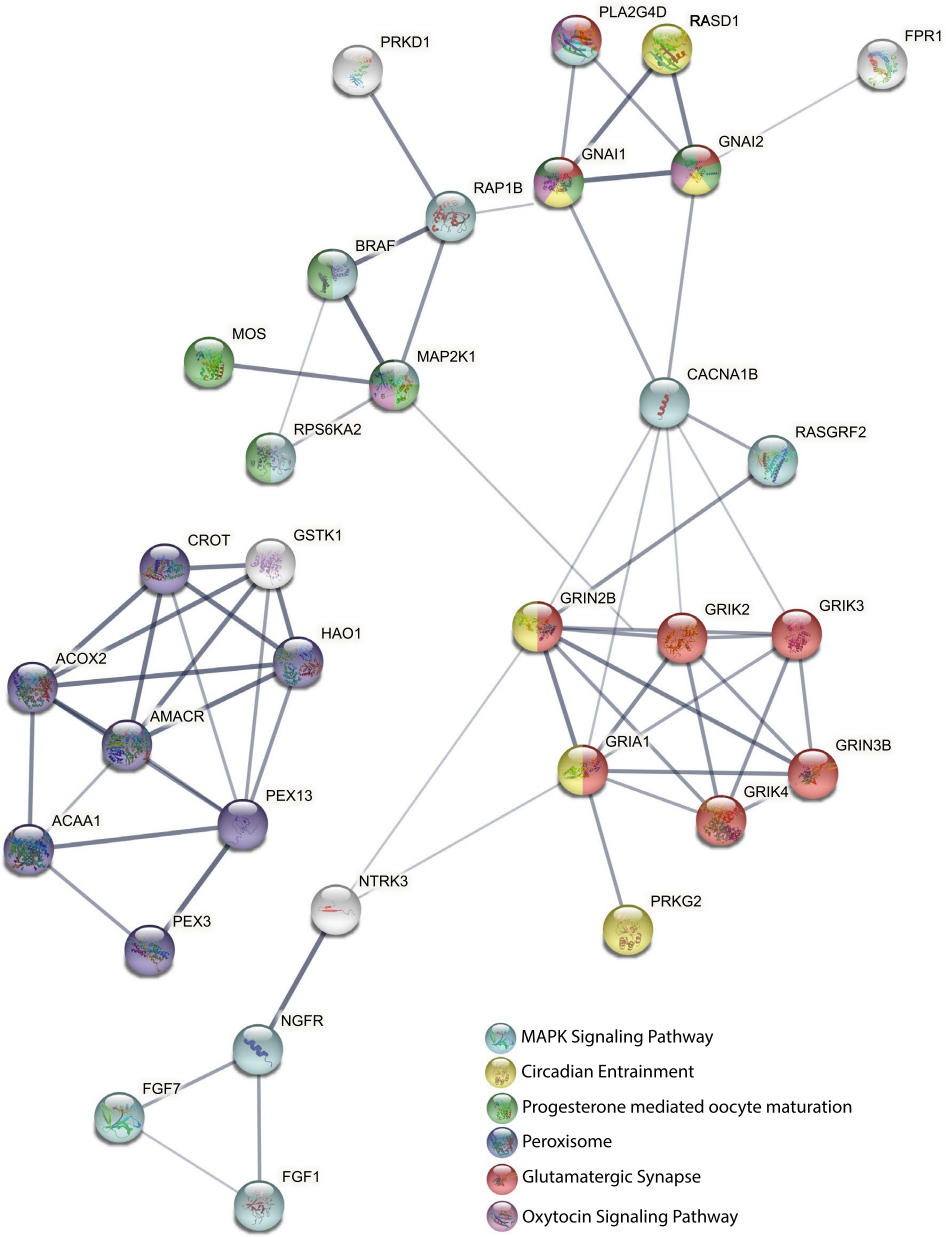
Protein–protein interaction network of DEGs. Color of the nodes indicates the pathway to which the genes belong. Edges show high confidence (0.9) interactions.

Protein–protein interaction network analysis of DEGs showed 32 genes to have strong interaction (minimum interaction score of 0.9). Genes functioning in MAPK signaling pathway, circadian entrainment, progesterone mediated oocyte maturation, glutamatergic synapse and oxytocin signaling pathways formed one cluster and genes that were part of peroxisome pathway formed a separate cluster (Fig. 6).

## Discussion

Genetic progress through selection for improved egg number or rate of lay have increased egg production in domestic birds particularly in chicken. The rapid development of various “omics” technologies has had a profound effect on animal breeding and have replaced classical breeding methods with molecular breeding, as these methods help in enhancing desired traits in animals at shorter generational intervals. RNA sequencing can help in deciphering the complex expression dynamics of genes under various physiological states. In poultry, as in all vertebrates, the reproductive endocrine systems and periodic ovulation are regulated by the hypothalamic–pituitary–gonadal axis. These regulatory mechanisms mediate physical interaction of follicles and directly contribute to egg-laying. Ovary development and follicle formation is a dynamic process and has a direct effect on egg-laying performance^14^, therefore identifying candidate genes that are differentially expressed in the ovaries of HEL and LEL ducks might increase the understanding of egg production rate in Chara ducks.

The HEL ducks had a significantly higher laying rate than the LEL ducks. On average the HEL ducks laid 433 eggs, while the LEL ducks laid only 221 eggs. Given this difference in egg laying, the transcriptome levels of the HEL ovary were compared against the LEL ovary, to identify genes and pathways that were differentially enriched. Overall, 489 genes were found to be differentially expressed out of which 310 were upregulated in the HEL ducks. This included 10 glutamatergic synapses related genes. Glutamate receptors mediate excitatory neurotransmission and increase LH and FSH secretion^15^. Several glutamatergic synapse genes were upregulated in the HEL ducks, including *GRIN2B, GRIK3, GRIA1 GRIK4* and *GRIK2*. The *GRIN2B,* (glutamate ionotropic receptor NMDA type subunit 2B), which acts as an agonist binding site for glutamate, and is reported to be a functional component for embryonic development^16^. *GRIK3* (Glutamate receptor ionotropic kainate 3), is a glutamate ionotropic receptor encoding gene associated with egg production in chickens^17^, and fertility in mouse^18^. *GRIA1* (Glutamate ionotropic receptor AMPA type subunit 1), which was found to influence ovulation rate and number of ova and embryos collected in super ovulated donors in cattle^19^. Previously, Chen et al.^14^ have also demonstrated the enrichment of glutamatergic synapses in the follicles of high laying chickens. This shows the vital role of glutamatergic synapses genes in embryonic development, egg production, fertility and reproduction.

Seven genes that are part of the progesterone mediated oocyte maturation were enriched among the DEGs and a few of these genes were also part of the MAPK (mitogen activated protein kinase) pathway. Both these pathways have been found to be associated with egg production in ducks^20,21^. MAPK signaling regulates diverse cellular mechanisms by relaying extracellular signals and activating intracellular response^22^ which is activated due to extracellular growth factor-receptor interactions. However, in female germ cells, it is activated by an upstream activator gene *MOS* (MOS proto-oncogene, serine/threonine kinase). *MOS* is a critical regulator of meiotic divisions that produces eggs^23^. *MOS* expression was found to be elevated in the ovary of the HEL ducks. Similarly, the expression of *BRAF* (B-Raf proto-oncogene, serine/threonine kinase) which is involved in progesterone mediated oocyte maturation and MAPK pathways, was also found to be elevated in the ovary of HEL ducks. *BRAF* has been previously reported to be associated with fertility and egg production in chickens^24^. The enrichment of these genes suggests increased meiotic division in the ovary of HEL ducks.

Several serine hydrolase, serine-type endopeptidase and serine-type peptidase genes were differentially enriched. Serine proteases have been reported to affect egg production, oocyte maturation and fertilization^12^. They have also been reported to be enriched in the ovary of high egg producing ducks^5^. Ovochymases are a serine active site protease and were found to be released during egg activation in *Xenopus laevis*^25^. Though their function is not clearly known in mammals or birds, Bourin et al.^26^ suggested that ovochymase-2 (*OVCH*2) might be secreted to be incorporated in the egg yolk or vitelline membrane. *OVCH2*, was found to be differentially expressed in the pituitary of high laying hen^12^ whereas *OVCH1* (Ovochymase-1) expression has not been previously reported in poultry. In our study, both *OVCH1* and *OVCH2* were upregulated in HEL ducks. Another group of genes highly enriched in HEL ducks were the cathepsin (CTS) family of lysosomal proteases, which were reported to be related to oviduct development in chickens^27^.

Several other genes related to egg production, hormone metabolism, and folliculogenesis were upregulated in the HEL ducks. This included *PAR6* (Par-6-family cell polarity regulator alpha), which is a major regulator of follicle number and follicle development^28^. *KPNA7* (Karyopherin subunit alpha 7) which functions as a receptor for oocyte-specific protein that are important for oocyte and embryonic development^29^. *HSD17B2* (Hydroxysteroid 17-beta dehydrogenase 2) a key regulator of steroid hormone metabolism^30^. *LHX9* (LIM homeobox 9), which plays a key role in female reproductive tract development^31^.

Notably several immune related genes were enriched in the LEL ducks. This included *Mx1* (Interferon-induced GTP binding protein), which plays an important role in antiviral activities by blocking viral transcription^32^. Integrins β family genes (*ITGB5* and *ITGB6*) mediate cell-to-extracellular matrix and cell-to-cell adhesions^33^. *ITGB5* plays a key role in triggering phagocytosis of apoptotic cells^34^, while *ITGB6* modulates innate immune response^33^. *MRC1* (Mannose receptor C-type 1) mediates clathrin dependent endocytosis and promotes antigen presentation and is expressed in response to infectious agents^35^. Interferon-stimulated gene 15 (*ISG15*) expression has been reported to inhibit the budding of retroviruses from cells^36^. The upregulation of these genes in the LEL ducks (downregulated in HEL ducks), possibly suggests that the immune system of the LEL ducks were under stress.

## Conclusions

Variation in egg production is likely caused by a combination of both genetic and environmental factors. This study identified several important pathways and candidate genes contributing to egg production in ducks. The difference in egg laying number between the HEL and LEL ducks could be a combination of increased expression of egg production and folliculogenesis related genes in HEL ducks and the increased expression of immune response related genes in the LEL ducks. These findings will have important implications for not only poultry industry but also for our understanding of adaptive variation in egg production in nature.

## Materials and methods

### Sample collection

The ovary samples used in the study were obtained from another study which was reviewed and approved by the Institutional Animal Ethics Committee of College of Veterinary and Animal Sciences, Kerala Veterinary and Animal Sciences University, Mannuthy, Thrissur, Kerala. This study is in accordance with ARRIVE guidelines. All methods were performed in accordance with relevant guidelines and regulations. Briefly, 300 Kuttanad ducks, the popular indigenous ducks of Kerala which includes Chara and Chemballi breeds were reared at the Poultry and Duck Farm, Kerala Veterinary and Animal Sciences University, Mannuthy, Thrissur, Kerala. At 20 weeks of age, 200 ducks were randomly selected and housed in individual cages with same feeding and management condition to vividly take into record the number of eggs being laid by each duck. Layer duck feed was provided ad libitum twice a day and water was supplied ad libitum through water trough. Egg number of individual ducks was recorded from 21 to 93 weeks of age. The ducks were sorted based on the total number of eggs laid at 93 weeks of age from high to low. The ducks with greater egg production (i.e. higher than 400 eggs in 93 weeks) were considered as high egg layers (HEL) and the ducks with lesser egg production (i.e. lower than 235 eggs in 93 weeks) were considered as low egg layers (LEL). We selected six and five Chara ducks from the HEL and LEL group respectively, for transcriptome sequencing. The average egg production rate was 433 ± 17 and 221 ± 9 for HEL and LEL ducks, respectively. Ducks were sacrificed during their laying physiological state through exsanguination. The ovary tissues including the follicles of different hierarchical grades were collected in RNA later solution after removing egg yolk and stored at − 80 °C.

### RNA isolation and sequencing

The total RNA extraction was performed using TRIzol method^37^. The ovarian tissue samples were washed briefly with a chilled 1X PBS buffer (10X PBS Buffer, Invitrogen, USA) before the extraction to avoid the antagonistic effect of RNA later in RNA yield. The quality and quantity of the RNA samples were assessed using NanoDrop (NanoDrop, Thermo Scientific) and Bioanalyzer (Agilent Technologies, USA). Approximately 4 µg of total RNA (RNA integrity number > 7) was used to prepare the cDNA library using TruSeq RNA Sample Prep Kits (Illumina, USA). Poly-A containing mRNA molecules were purified using poly-T oligo-attached magnetic beads and fragmented using divalent cations. The cleaved RNA fragments were used to synthesise first strand cDNA using reverse transcriptase and random hexamer primers followed by second strand cDNA synthesis. Subsequently, the synthesised cDNA fragments were then processed for end repairing followed by addition of single ‘A’ base and adapter ligation. The products were purified and enriched with PCR to create the sequencing cDNA library. The paired end libraries were sequenced on Illumina HiSeq 2500 platform (Illumina, USA).

### Quantitative real time RT-PCR

To validate the RNA-Seq results, the expression of seven randomly selected DEGs were assessed using qRT-PCR. Total RNA was extracted from ovary samples using TRIzol Kit (Invitrogen). The primers (Supplementary Table 5) were designed using Primer 3 software (https://primer3.ut.ee/). PCR was performed using Light Cycler 480 SYBR Green I Master on a Roche Light Cycler 480 System. The relative gene expression levels of selected DEGs were quantified based on β*-actin* gene expression by 2^−ΔΔ*Ct*^ method.

### Transcriptome data analysis

The raw transcriptome data comprising reads with adaptor sequences, sequencing primers and low-quality reads were processed using cutadapt^38^ to obtain high quality clean reads. Subsequently, the clean reads were aligned and mapped to the reference duck genome (CAU_duck1.0) using the STAR v2.7.2b aligner^39^. The genome annotation was downloaded from Ensembl (http://ftp.ensembl.org/pub/release-104/gtf/anas_platyrhynchos_platyrhynchos/Anas_platyrhynchos_platyrhynchos.CAU_duck1.0.104.gtf.gz)). Mapped reads were counted using HTSeq v0.9.1^40^. The raw counts were processed and analysed using the DESeq2 v1.28.1 package^41^ in R version 4.0.2 (https://www.R-project.org) following the methods previously described^42^. The normalized count data were used for all the downstream analysis. Principal Component Analysis (PCA) was performed by calculating the distance between samples using the *vst* function in DESeq2. The *p* values were adjusted using the Benjamini–Hochberg method^43^ and genes with an absolute fold change > 1.5 (absolute log2FC > 0.5), *p* < 0.005 and false discovery rate (FDR) < 0.1 were considered as significantly differentially expressed (DEG). Duck Ensembl gene IDs were converted to human orthologs, and the matched official gene symbols were used for functional clustering and pathway analysis using the Database for Annotation, Visualization and Integrated Discovery (DAVID)^44^. Annotation was performed under Biological Process (BP), Molecular Function (MF), Cellular Component (CC) in the InterPro protein domain and KEGG (Kyoto Encyclopedia of Genes and Genomes) pathway databases^45^. Significant terms (FDR < 0.1) were plotted using the ggplot2 package in R version 4.0.2. Protein–protein interaction network analysis of the DEGs was performed using search tool for the retrieval of interacting genes/proteins (STRING)^46^ and the resulting interaction network was visualized using Cytoscape v.3.9.0^47^.

## Supplementary Information

Supplementary Tables.

## Data Availability

The transcriptome data raw files have been deposited at Sequence Read Archive (GenBank) under the BioProject ID PRJNA825149.

## References

[1] Patil SS (2021). A systematic review and meta-analysis on the prevalence of infectious diseases of Duck: A world perspective. Saudi J. Biol. Sci..

[2] Veeramani P, Prabakaran R, Sivaselvam SN, Sivakumar T, Selvan ST, Karthickeyan SMK (2016). Phylogenetic analysis of six duck populations. Indian J. Anim. Res..

[3] George TG, Nayar R, Cyriac S (2012). Yields and ratios of different meat parts of Vigova Super M and Kuttanad Ducks: A comparison. Int. J. Sci. Res..

[4] Bédécarrats GY, McFarlane H, Maddineni SR, Ramachandran R (2009). Gonadotropin-inhibitory hormone receptor signaling and its impact on reproduction in chickens. Gen. Comp. Endocrinol..

[5] Tao Z (2017). Comparative transcriptomic analysis of high and low egg-producing duck ovaries. Poult. Sci..

[6] Sun Y (2020). Identification of differentially expressed genes and signaling pathways in the ovary of higher and lower laying ducks. Br. Poult. Sci..

[7] Bao X (2021). Comparative transcriptome profiling of ovary tissue between black muscovy duck and white Muscovy duck with high-and low-egg production. Genes.

[8] Bello FS (2021). Hypothalamic and ovarian transcriptome profiling reveals potential candidate genes in low and high egg production of white Muscovy ducks (*Cairina moschata*). Poult. Sci..

[9] Ouyang Q (2020). Comparative transcriptome analysis suggests key roles for 5-hydroxytryptamlne receptors in control of goose egg production. Genes.

[10] Mu R (2021). Transcriptome analysis of ovary tissues from low-and high-yielding Changshun green-shell laying hens. BMC Genom..

[11] Zhang Q (2021). Comparative transcriptomic analysis of ovaries from high and low egg-laying Lingyun black-bone chickens. Vet. Med. Sci..

[12] Wang C, Ma W (2019). Hypothalamic and pituitary transcriptome profiling using RNA-sequencing in high-yielding and low-yielding laying hens. Sci. Rep..

[13] Hu Z (2021). Skeletal muscle transcriptome analysis of Hanzhong Ma Duck at different growth stages using RNA-Seq. Biomolecules.

[14] Chen X (2021). Transcriptome analysis of ovarian follicles reveals potential pivotal genes associated with increased and decreased rates of chicken egg production. Front. Genet..

[15] Gill S, Barker M, Pulido O (2008). Neuroexcitatory targets in the female reproductive system of the nonhuman primate *(Macacafascicularis*). Toxicol. Pathol..

[16] Seki R (2017). Functional roles of Aves class-specific cis-regulatory elements on macroevolution of bird-specific features. Nat. Commun..

[17] Yuan J (2015). Identification of promising mutants associated with egg production traits revealed by genome-wide association study. PLoS ONE.

[18] Sominsky L, Goularte JF, Andrews ZB, Spencer SJ (2019). Acylated ghrelin supports the ovarian transcriptome and follicles in the mouse: Implications for fertility. Front. Endocrinol..

[19] Sugimoto M (2010). Ionotropic glutamate receptor AMPA 1 is associated with ovulation rate. PLoS ONE.

[20] Qiu M (2020). High-throughput sequencing analysis identified microRNAs associated with egg production in ducks ovaries. Peer J..

[21] Zou K (2020). Ovarian transcriptomic analysis and follicular development of Leizhou black duck. Poult. Sci..

[22] Cargnello M, Roux PP (2011). Activation and function of the MAPKs and their substrates, the MAPK-activated protein kinases. Microbiol. Mol. Biol. Rev..

[23] Dupré A, Haccard O, Jessus C (2011). Mos in the oocyte: How to use MAPK independently of growth factors and transcription to control meiotic divisions. J. Signal Transduct.

[24] Yang L (2021). Transcriptome analysis and identification of age-associated fertility decreased genes in hen uterovaginal junction. Poult. Sci..

[25] Lindsay LL, Yang JC, Hedrick JL (1999). Ovochymase, a Xenopus laevis egg extracellular protease, is translated as part of an unusual polyprotease. Proc. Natl. Acad. Sci..

[26] Bourin M (2012). Transcriptomic profiling of proteases and antiproteases in the liver of sexually mature hens in relation to vitellogenesis. BMC Genom..

[27] Jeong W (2012). AHCYL1 is mediated by estrogen-induced ERK1/2 MAPK cell signaling and microRNA regulation to effect functional aspects of the avian oviduct. PLoS ONE.

[28] Wen J, Zhang H, Li G, Mao G, Chen X (2009). PAR6, a potential marker for the germ cells selected to form primordial follicles in mouse ovary. PLoS ONE.

[29] Hu J (2010). Novel importin-α family member Kpna7 is required for normal fertility and fecundity in the mouse. J. Biol. Chem..

[30] Ge K, Chen X, Kuang J, Yang L, Geng Z (2019). Comparison of liver transcriptome from high-and low-intramuscular fat Chaohu ducks provided additional candidate genes for lipid selection. 3 Biotech.

[31] Birk OS (2000). The LIM homeobox gene Lhx9 is essential for mouse gonad formation. Nature.

[32] Wang Y, Lupiani B, Reddy SM, Lamont SJ, Zhou H (2014). RNA-seq analysis revealed novel genes and signaling pathway associated with disease resistance to avian influenza virus infection in chickens. Poult. Sci..

[33] Koivisto L, Bi J, Häkkinen L, Larjava H (2018). Integrin αvβ6: Structure, function and role in health and disease. Int. J Biochem. Cell Biol..

[34] Singh S, D’mello V, Henegouwen BP, Birge BR (2007). A NPxY-independent β5 integrin activation signal regulates phagocytosis of apoptotic cells. Biochem. Biophys. Res. Commun..

[35] Wattrang E (2020). Immune responses upon experimental *Erysipelothrix rhusiopathiae* infection of naïve and vaccinated chickens. Vet. Res..

[36] Kuang Z, Seo EJ, Leis J (2011). Mechanism of inhibition of retrovirus release from cells by interferon-induced gene ISG15. J. Virol..

[37] Nimisha K (2022). Comparative liver transcriptome analysis of duck reveals potential genes associated with egg production. Mol. Biol. Rep..

[38] Martin M (2011). Cutadapt removes adapter sequences from high-throughput sequencing reads. EMBnet. J..

[39] Dobin A (2013). STAR: Ultrafast universal RNA-seq aligner. Bioinformatics.

[40] Anders S, Pyl PT, Huber W (2015). HTSeq-a Python framework to work with high-through put sequencing data. Bioinformatics.

[41] Love MI, Huber W, Anders S (2014). Moderated estimation of fold change and dispersion for RNA-seq data with DESeq2. Genome Biol..

[42] Srikanth K (2019). Cardiac and skeletal muscle transcriptome response to heat stress in Kenyan chicken ecotypes adapted to low and high altitudes reveal differences in thermal tolerance and stress response. Front. Genet..

[43] Benjamini Y, Hochberg Y (1995). Controlling the false discovery rate: A practical and powerful approach to multiple testing. J. R. Stat. Soc. B (Methodol.).

[44] Huang DW, Sherman BT, Lempicki RA (2008). Systematic and integrative analysis of large gene lists using DAVID bioinformatics resources. Nat. Protoc..

[45] Kanehisa M, Goto S (2000). KEGG: Kyoto encyclopedia of genes and genomes. Nucleic Acids Res..

[46] Szklarczyk D (2015). STRING v10: Protein–protein interaction networks, integrated over the tree of life. Nucleic Acids Res..

[47] Shannon P (2003). Cytoscape: A software environment for integrated models of biomolecular interaction networks. Genome Res..

